# The profile of gastrointestinal dysfunction in prodromal to late-stage Parkinson’s disease

**DOI:** 10.1038/s41531-025-00900-9

**Published:** 2025-05-11

**Authors:** Marta Camacho, Julia C. Greenland, Cyrus Daruwalla, Kirsten M. Scott, Bina Patel, Dimitrius Apostolopoulos, Joana Ribeiro, Molly O’Reilly, Michele T. Hu, Caroline H. Williams-Gray

**Affiliations:** 1 Department of Clinical Neurosciences, University of Cambridge, Cambridge, UK; 2 Cambridge University Hospitals NHS Foundation Trust, Cambridge, UK; 3 Nuffield Department Clinical Neurosciences, University of Oxford, Oxford, UK

**Keywords:** Parkinson's disease, Neurological manifestations, Epidemiology

## Abstract

Gastrointestinal dysfunction (GID) may play a key role in Parkinson’s disease (PD) but its relationship with disease progression remains unclear. We recruited 404 PD cases, 37 iRBD (isolated REM Sleep Behaviour Disorder) and 105 controls. Participants completed the Gastrointestinal Dysfunction Scale for PD (GIDS-PD) and standardised disease severity assessments. Whole gut transit time (WGTT) was measured by ingestion of blue dye and recorded time to blue stools appearance (‘Blue Poop Challenge’) in a subset of PD cases. Gastrointestinal symptoms were more common and prevalent in iRBD and PD versus controls, and WGTT was significantly higher in PD versus controls. After adjustment for confounding factors, disease stage was not a significant predictor of GIDS-PD Constipation or Bowel Irritability scores. Longitudinal assessment of GIDS-PD scores and WGTT confirmed stability over a 4 year period. Bowel dysfunction may be a phenotypic feature in a subset of Parkinson’s with implications for patient stratification and management.

## Introduction

Although it is widely accepted that gastrointestinal (GI) symptoms are common in Parkinson’s disease (PD), there is a lack of information on how GI dysfunction (GID) changes over the disease course. Given that constipation has been identified as a risk factor for faster PD progression^1^ and a feature of a proposed PD subtype^2^, and that dysphagia and weight loss are associated with accelerated mortality^3,4^, it is important to identify how early in the disease process these symptoms begin, how prevalent they are and whether they are aggravated or ameliorated by disease severity and other factors such as medication and lifestyle.

Classically, constipation is thought to increase with Parkinson’s severity and studies have previously reported an association between increasing disease severity/duration and constipation but showed only modest correlations or did not present the strength of the association^5,6^. Furthermore, longitudinal studies of GID in PD^7–10^ have reported an increase in symptoms over time but most exhibit important methodological limitations such as small and heterogeneous clinical samples, the use of limited questions or general non-motor instruments to assess GID, and typically fail to account for relevant clinical and lifestyle factors such as medication, diet, water intake and exercise.

Moreover, most psychometric tools used to assess GID in PD have null or modest correlations with objective measures of GID^11–13^. Currently available objective measures of gut motility include magnetic resonance imaging^14^, scintigraphy^15^, radio opaque markers^16^ and wireless motility capsules^17^. These techniques are expensive, invasive and warrant in-person attendance at a specialised centre, requiring specialised equipment and staff, which prevents their widespread use in studies. The ingestion of blue dye, colloquially named ‘Blue Poop Challenge’ (BPC), is a safe, an inexpensive and scalable technique to measure whole gut transit time (WGTT), has been validated in a large cohort of healthy individuals and found to be the strongest marker of transit time compared to traditional psychometric transit time measures^18^. However, the BPC has not previously been employed to characterise WGTT in PD.

In this study, we used a PD-specific validated self-report questionnaire, the Gastrointestinal Dysfunction for Parkinson’s Disease Scale (GIDS-PD)^19^ to characterise the profile of gastrointestinal symptoms across PD stages, from RBD to late-stage disease, in order to better understand its heterogeneity, and assessed longitudinal change in GIDS-PD scores over time, adjusting for relevant clinical confounders. We also employed the BPC in PD to objectively assess gut transit time over the disease course.

## Results

### Demographic and clinical description of PD sample

A total of 404 people with PD (H&Y = 1, *n* = 77; H&Y = 2, *n* = 234; H&Y = 3, *n* = 57; H&Y = 4, *n* = 34; H&Y5 = 2), 37 people with iRBD and 72 controls participated in the study. A summary of demographic and clinical sample characteristics is presented in Table 1. When comparing across PD stages, there were no statistical differences in prevalence of iRBD history, diet, water intake and smoking. There were no differences in age, MoCA scores, opioid-based drugs and anticholinergic drugs, diet, water intake and smoking between the PD, iRBD and control groups. There were also no differences in self-reported GI diagnoses between groups except for constipation, with higher prevalences in the iRBD and PD compared to the control group. One iRBD participant was on a dopamine agonist for Restless Legs Syndrome. There was a statistically significant increase in age, disease duration, MDS-UPDRS scores, LEDD, opioid-based drugs and anticholinergic drugs across disease stage, whilst MoCA scores decreased, denoting worse disease severity across the H&Y stages.

**Table 1:** Demographic and clinical characteristics of PD patients stratified by H&Y stage, RBD cases, and controls

	Controls (*n* = 105)	RBD (*n* = 37)	PD H&Y = 1 (*n* = 77)	PD H&Y = 2 (*n* = 234)	PD H&Y = 3 (*n* = 57)	PD H&Y = 4+ (*n* = 36)	*p* value
Age at visit (years)	68.5 ± 8.1 (46–86)	70.2 ± 6.7 (51–82)	62.9 ± 9.9 (40–85)	67.7 ± 8.7 (36–86)	71.8 ± 8.0 (49–83)	76.5 ± 6.8 (59–89)	0. ^a^ < 0.001^b^*
Sex (% males)	50.5%	94.6%	66.2%	69.2%	61.4%	50.0%	<0.001^a^* 0.126 ^b^
Education (years)	13.9 ± 3.2 (10–21)	13.4 ± 2.9 (10–20)	14.4 ± 3.5 (6–23)	13.7 ± 3.0 (9–23)	12.7 ± 2.6 (10–18)	13.1 ± 3.1 (10–22)	0.665^a^ 0.007^b^*
Time from RBD/PD diagnosis (years)	–	5.6 ± 2.8 (1–12)	1.5 ± 2.3 (0–12)	3.0 ± 3.2 (0–12)	6.1 ± 5.4 (0–21)	11.5 ± 4.7 (2–21)	<0.001^b^*
Positive RBD history prior to PD diagnosis	–	–	22.6%	40.6%	35.7%	33.3%	0.146^b^
MDS-UPDRS total score	–	–	31.5 ± 10.8 (7–62)	52.7 ± 16.9 (19–134)	80.2 ± 19.3 (39–127)	108.4 ± 19.4 (77–170)	<0.001^b^*
MDS-UPDRS part I	–	8.5 ± 5.4 (0–25)	7.6 ± 4.2 (0–19)	9.6 ± 5.2 (0–30)	13.4 ± 5.9 (4–28)	15.9 ± 6.8 (3–36)	0.077^a^ <0.001^b^*
MDS-UPDRS II	–	2.8 ± 3.2 (0–12)	5.7 ± 3.6 (0–16)	10.2 ± 5.2 (1–32)	18.2 ± 9.4 (0–60)	28.3 ± 6.9 (16–42)	<0.001^a^ <0.001^b^*
MDS-UPDRS part III	–	7.8 ± 4.3 (0–18)	17.8 ± 6.9 (5–38)	31.7 ± 10.5 (10–62)	46.0 ± 12.6 (19–75)	59.4 ± 13.1 (34–91)	<0.001^a^* <0.001^b^*
MDS-UPDRS part IV	–	–	0.5 ± 1.2 (0–5)	1.5 ± 2.6 (0–13)	3.3 ± 4.1 (0–17)	5.0 ± 4.4 (0–18)	<0.001^b^*
MoCA total score	–	26.1 ± 2.1 (22–30)	26.5 ± 2.4 (20–30)	26.0 ± 2.7 (19–30)	25.0 ± 2.7 (20–30)	24.8 ± 2.8 (19–29)	0.921^a^ 0.002^b^*
LEDD (mg)	–	1.1 ± 6.6 (0–40)	276.9 ± 198.3 (0–994)	397.7 ± 316.9 (0–1857)	641.4 ± 444.7 (0–2195)	1102.9 ± 540.8 (300–2980)	<0.001^a^* <0.001^b^*
Anticholinergic drugs (%)	–	10.8%	13.0%	11.6%	31.6%	41.7%	0.219^a^ <0.001^b^*
Opiate-based drugs (%)	–	0%	6.5%	3.0%	3.6%	22.2%	0.145^a^ <0.001^b^*
Diet (Vegetarian, %)	1.9%	10.8%	6.5%	4.3%	7.0%	2.9%	0.088^a^ 0.295^b^
GIDS-PD Water intake (Less than 1 glass a day, %)	20.0%	21.6%	14.3%	15.8%	15.8%	8.3%	0.81^a^ 0.696^b^
GIDS-PD Exercise (Less than once a week, %)	24.8%	32.5%	18.2%	29.6%	47.4%	58.3%	0.295^a^ <0.001^b^*
GIDS-PD Smoking (% current smokers)	61.9%	48.6%	58.4%	58.4%	57.9%	52.8%	0.333^a^ 0.937^b^
GIDS-PD Caffeine intake (less 1 cup/day)	7.6%	8.1%	20.8%	18.0%	14.0%	38.9%	0.004^a^* 0.019^b^*
GIDS-PD Laxative use (% daily laxative use)	7.6%	5.4%	5.3%	15.0%	29.6%	25.0%	<0.001^a^* <0.001^b^*
Constipation diagnosis (self-reported)	5.7%	21.6%	7.8%	17.1%	31.6%	50.0%	0.002 ^a^* <0.001^b^*
IBS diagnosis (self-reported)	9.5%	2.7%	13.0%	5.6%	7.0%	8.3%	0.397^a^ 0.194^b^
GORD diagnosis (self-reported)	19.0%	27.0%	13.0%	13.7%	24.6%	27.8%	0.239 ^a^ 0.044^b^*
Colon/Bowel Cancer (self-reported)	1.9%	0%	0%	0.4%	1.8%	0%	0.277 0.490^b^

Significance threshold is *p* < 0.05 and illustrated by *. Values shown as mean ± SD (range).

*GIDS-PD* Gastrointestinal Dysfunction Scale for Parkinson’s disease, *MDS-UPDRS* Movement Disorders Society-Unified Parkinson’s Disease Rating Scale, *RBD* REM Sleep Behaviour Diagnosis, *MoCA* Montreal Cognitive Assessment, *IBS* Irritable Bowel Syndrome, *GORD* Gastroesophageal reflux disease.

^a^Comparisons between control, iRBD and PD groups.

^b^Comparisons between PD stages H&Y1 through 4+.

### GID symptoms across disease stage

**Fig. 1:**
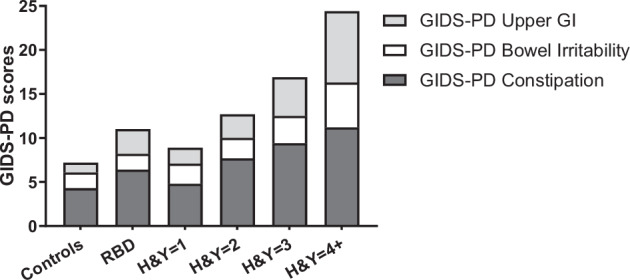
Mean GIDS-PD scores in controls, RBD and across H&Y stages. Unadjusted GIDS-PD Constipation, Bowel Irritability and Upper GI domain scores increased across disease stage. GIDS-PD Gastrointestinal Dysfunction Scale for Parkinson’s disease, H&Y Hoehn and Yahr, RBD REM Sleep Behaviour disorder.

Table 2 summarises individual and composite GIDS-PD scores across the groups. Unadjusted comparisons of GIDS-PD domain scores across the PD groups, stratified by H&Y stage, demonstrated a main effect of PD stage on GIDS-PD Constipation scores (*H*(3) = 36.714, *p* < 0.001), Bowel Irritability (*H*(3) = 11.402, *p* = 0.010) and Upper GI scores (*H*(3) = 50.580, *p* < 0.001). This indicates a general increase in self-reported GI symptoms in all domains with advancing disease stage (Fig. 1). Accordingly, we found a correlation between motor disease severity (MDS-UPDRS-III) and both GIDS-PD Constipation score (rho = 0.19, *p* < 0.001) and GIDS-PD Upper GI score (rho = 0.25, *p* < 0.0001).

**Table 2:** Gastrointestinal symptom prevalence and mean scores of GIDS-PD items and domain scores across disease stages

GIDS-PD	Controls (*n* = 105)	RBD (*n* = 37)	PD H&Y = 1 (*n* = 77)	PD H&Y = 2 (*n* = 234)	PD H&Y = 3 (*n* = 57)	PD H&Y = 4+ (*n* = 36)	*p* value
1. Low Bowel Emptying Frequency (3 times a week or less)	8.6%	35.1%	19.5%	42.3%	64.3%	69.4%	
	2.0 ± 1.2 (1–9)	2.8 ± 1.8 (1–9)	2.2 ± 1.3 (1–6)	3.2 ± 2.2 (1–9)	3.7 ± 2.2 (1–9)	4.0 ± 2.0 (1–9)	<0.001^a^* < 0.001^b^*
Employed methods to increase stool frequency	17.1%	32.4%	27.6%	44.2%	63.0%	73.0%	0.231^a^ <0.001^b^*
2. Straining	38.1%	67.6%	33.8%	59.0%	63.2%	72.2%	
	0.8 ± 1.3 (0–6)	1.5 ± 1.7 (1–6)	0.8 ± 1.4 (0–6)	1.7 ± 2.3 (0–9)	2.0 ± 2.4 (0–9)	2.7 ± 2.5 (0–9)	0.001^a^* < 0.001^b^*
3. Hard stools	50.5%	32.4%	46.8%	64.1%	64.9%	69.4%	
	1.0 ± 1.4 (0–6)	1.5 ± 1.5 (0–6)	1.0 ± 1.5 (0–6)	1.7 ± 2.1 (0–9)	1.9 ± 2.3 (0–9)	2.2 ± 2.2 (0–9)	0.020^a^* 0.003^b^*
4. Incomplete evacuation	32.4%	35.1%	44.2%	51.1%	62.5%	72.2%	
	0.5 ± 0.9 (0–6)	0.7 ± 1.2 (0–4)	0.9 ± 1.4 (0–6)	1.1 ± 1.7 (0–9)	1.7 ± 2.4 (0–9)	2.3 ± 2.7 (0–9)	0.001^a^* 0.001^b^*
5. Abdominal pain	20.0%	16.2%	27.3%	20.9%	32.1%	48.6%	
	0.5 ± 0.9 (0–9)	0.2 ± 0.5 (0–2)	0.4 ± 0.8 (0–3)	0.5 ± 1.4 (0–9)	0.8 ± 1.6 (0–6)	1.3 ± 2.3 (0–9)	0.153^a^ 0.003^b^*
6. Increased stool passing	27.6%	32.4%	31.6%	24.5%	37.5%	44.4%	
	0.5 ± 1.4 (0–9)	0.7 ± 1.3 (0–4)	0.7 ± 1.6 (0–9)	0.5 ± 1.0 (0–6)	1.0 ± 1.7 (0–6)	1.2 ± 2.3 (0–9)	0.734^a^ 0.020^b^*
7. Abdominal fullness	39.1%	43.2%	39.5%	47.9%	39.3%	55.6%	
	0.8 ± 1.5 (0–9)	0.7 ± 1.2 (0–4)	1.1 ± 1.8 (0–6)	1.1 ± 1.8 (0–9)	1.0 ± 1.7 (0–9)	1.8 ± 2.5 (0–9)	0.325^a^ 0.190^b^
8. Involuntary weight loss	5.7%	5.4%	15.8%	17.1%	27.3%	36.1%	
	0.1 ± 0.2 (0–1)	0.1 ± 0.4 (0–2)	0.2 ± 0.5 (0–3)	0.3 ± 0.8 (0–6)	0.3 ± 0.6 (0–2)	1.3 ± 2.8 (0–9)	<0.001^a^* 0.012^b^*
9. Difficulty swallowing	6.7%	24.3%	16.0%	29.9%	45.6%	63.9%	
	0.1 ± 0.3 (0–2)	0.6 ± 1.3 (0–6)	0.2 ± 0.5 (0–3)	0.6 ± 1.3 (0–9)	1.2 ± 1.9 (0–9)	2.7 ± 3.2 (0–9)	<0.001^a^* < 0.001^b^*
10. Excessive saliva	5.7%	18.9%	30.3%	44.0%	56.1%	83.3%	
	0.1 ± 0.7 (0–6)	0.8 ± 2.1 (0–9)	0.6 ± 1.1 (0–6)	1.2 ± 1.9 (0–9)	2.0 ± 2.6 (0–9)	3.6 ± 2.8 (0–9)	<0.001^a^* < 0.001^b^*
11. Heartburn	33.3%	48.6%	27.6%	25.2%	26.3%	44.4%	
	0.7 ± 1.4 (0–9)	1.3 ± 1.8 (0–6)	0.6 ± 1.2 (0–6)	0.5 ± 1.0 (0–6)	0.8 ± 1.6 (0–6)	1.1 ± 1.8 (0–9)	0.009^a^* 0.082^b^
12. Nausea	13.3%	10.0%	23.4%	21.8%	21.1%	30.6%	
	0.2 ± 0.6 (0–4)	0.1 ± 0.5 (0–2)	0.4 ± 1.1 (0–6)	0.4 ± 1.2 (0–9)	0.5 ± 1.2 (0–6)	0.9 ± 1.9 (0–9)	0.016^a^* 0.647^b^
GIDS-PD Total score	7.1 ± 6.7 (1–28)	11.0 ± 7.9 (1–34)	9.0 ± 8.4 (1–39)	12.6 ± 10.7 (1–68)	16.9 ± 12.7 (2–57)	25.0 ± 16.8 (2–78)	<0.001^a^* < 0.001^b^*
GIDS-PD Constipation score	4.3 ± 3.8 (1–23)	6.4 ± 5.0 (1–18)	4.8 ± 4.9 (1–24)	7.7 ± 7.0 (1–36)	9.4 ± 8.1 (1–36)	11.2 ± 6.7 (2–28)	<0.001^a^* < 0.001^b^*
GIDS-PD Bowel Irritability score	1.8 ± 3.4 (0–18)	1.8 ± 1.9 (0–6)	2.3 ± 3.4 (0–18)	2.3 ± 3.6 (0–22)	3.1 ± 4.4 (0–18)	5.1 ± 6.1 (0–24)	0.045^a^ 0.010^b^*
GIDS-PD Upper GI score	1.1 ± 1.9 (0–9)	2.8 ± 3.5 (0–15)	1.8 ± 2.7 (0–14)	2.7 ± 3.2 (0–16)	4.4 ± 3.9 (0–15)	8.1 ± 6.9 (0–27)	<0.001^a^* < 0.001^b^*

Prevalence of each item based on percentage of participants scoring 1 or more (except for bowel movement prevalence which was based on a score of 3 or more). Mean ± SD (range) scores for each item GIDS-PD domain scores are shown.

*H&Y* Hoehn and Yahr, *PD* Parkinson’s disease, *RBD* REM Sleep Behaviour Disorder, *GIDS-PD* Gastrointestinal Dysfunction Scale for Parkinson’s disease.

^a^Comparisons between control, iRBD and PD groups.

^b^Comparisons between PD stages H&Y1 through 4+.

GIDS-PD Constipation, Bowel Irritability and Upper GI scores were higher in the iRBD group and PD compared to controls, but not between PD and iRBD, suggesting that the GIDS-PD is sensitive to early GI dysfunction in prodromal states. Interestingly, the iRBD group also had higher GIDS-PD Constipation than early PD (H&Y = 1), but this significance was lost after Bonferroni correction. In terms of frequency of individual symptoms, hard stools and incomplete sense of evacuation were the most reported symptoms across groups (Table 2). Interestingly, difficulty swallowing was also common across groups, with a higher prevalence in iRBD cases compared to early PD (H&Y = 1) (Table 2).

In the PD group, participants with a positive RBD history prior to PD diagnosis had significantly higher GIDS-PD Constipation scores (9.4 ± 8.0) compared to those with a negative RBD history (7.0 ± 6.4; Mann–Whitney: *U* = 8013.0, *p* = 0.016).

In the PD group, linear regression analysis was performed to further evaluate the effect of disease stage on gastrointestinal dysfunction with adjustment for relevant covariates. This showed that H&Y stage was predictive of GIDS-PD Upper GI scores but not GIDS-PD Constipation and Bowel irritability domain score (Table 3). Having a positive history of prodromal RBD was the strongest predictor of GIDS-PD Constipation scores. Exercising 3 times a week or more was also associated with decreased Constipation scores. Caffeine intake was associated with lower GIDS-PD Bowel Irritability scores and anticholinergic medication use was associated with higher Bowel Irritability scores. GIDS-PD Upper GI scores were associated with higher H&Y stage and use of anticholinergic medication. We also repeated regression analysis with MDS-UPDRS III instead of H&Y for all GIDS-PD subscores and confirmed our results, MDS-UPDRS scores were a significant predictor for GIDS-PD Upper GI scores but not Constipation or Bowel Irritability (Supplementary Table 1).

**Table 3:** Regression models for GIDS-PD domain scores

**GIDS-PD constipation**	**Model 1** ***R***^2^ **=** **0.08** ***(F*(3, 398)** **=** **10.734,** ***p*** **<** **0.001)**			**Model 2** ***R***^2^ **=** **0.16** **(*F*(12, 227)** **=** **3.457,** ***p*** **<** **0.001)**		
	**β**	**95% CI**	***p*** **value**	**β**	**95% CI**	***p*** **value**
H&Y	2.05	1.16–2.94	<0.001*	0.99	−0.34–2.29	0.143
Age at visit	−0.05	−0.08–0.07	0.893	0.16	−0.09–0.12	0.762
Sex (Female)	1.74	0.33–3.15	0.016*	1.63	−0.24–3.49	0.087
Exercise (3+ times a week)				−1.20	−2.29–0.11	0.031*
Anticholinergic use				2.23	−0.42–4.87	0.099
Opioids				−3.81	−7.73–0.11	0.057
Prodromal RBD				2.17	0.30–4.05	0.023*
LEDD				0.00	−0.001–0.005	0.228
Vegetarian diet				2.95	−0.72–6.62	0.114
Water (4+ a day)				0.31	−1.11–1.73	0.670
Low caffeine (<1 caffeinated drink a day)				0.61	−1.64–2.85	0.531
Smoking (never smoked)				−0.61	−2.41–1.18	0.502
**GIDS-PD Bowel irritability**	**Model 1** ***R***^2^ **=** **0.05 (*F*(3, 395)** **=** **6.741,** ***p*** **<** **0.001)**			**Model 2** ***R***^2^ **=** **0.19 (*F*(12, 223)** **=** **4.282,** ***p*** **<** **0.001)**		
	**β**	**95% CI**	***p*** **value**	**β**	**95% CI**	***p*** **value**
H&Y	0.92	0.40–1.43	<0.001*	0.58	−0.17–1.34	0.127
Age at visit	−0.03	−0.08– 0.01	0.142	−0.04	−0.10–0.02	0.165
Sex (Female)	1.02	0.21–1.84	0.014*	0.92	−0.14–1.98	0.087
Caffeine (4+ cups a day)				−1.04	−1.74–0.34	0.004*
Smoking (Never smoked)				−0.95	−1.98–0.09	0.072
Low Exercise (less than once a week)				0.91	−0.17–1.98	0.098
Anticholinergic use				1.80	−0.28–3.32	0.021*
Opioids				−0.48	−2.70–1.73	0.668
LEDD				0.00	0.0–0.003	0.143
Vegetarian diet				−1.09	−3.16–0.98	0.300
Water intake (4+ a day)				0.69	−0.12–1.49	0.096
RBD history				0.07	−0.99–1.14	0.891
**GIDS-PD** **Upper GI**	**Model 1** ***R***^2^ **=** **0.16 (*F*(3, 399)** **=** **24.517,** ***p*** **<** **0.001)**			**Model 2** ***R***^2^ **=** **0.23 (*F*(9, 232)** **=** **7.695,** ***p*** **<** **0.001)**		
	**β**	**95% CI**	***p*** **value**	**β**	**95% CI**	***p*** **value**
H&Y	1.94	1.46–2.43	<0.001*	1.79	1.08–1.08	<0.001*
Age at visit	0.00	−0.04–0.04	0.999	0.01	−0.04–0.07	0.645
Sex (Female)	0.00	−0.77–0.77	0.995	0.17	−0.84–1.17	0.743
LEDD				0.0	0.0–0.002	0.159
Anticholinergic use				1.47	−0.12–2.83	0.033*
Water intake (4+ a day)				0.42	−0.34–1.19	0.276
Caffeine (4+ cups a day)				−0.34	−0.99–0.316	0.310
Exercise (3+ times a week)				0.55	−0.04–1.13	0.067
RBD history				0.46	−0.55–1.47	0.367

Significance threshold *p* < 0.05, represented by *.

*β* beta coefficient, *CI* confidence interval, *PD* Parkinson’s disease, *GIDS-PD* Gastrointestinal Dysfunction Scale for Parkinson’s disease, *LEDD* Levodopa equivalent daily dose, *H&Y* Hoehn and Yahr.

Because the prevalence of daily laxative use increased significantly across stages and including it as a covariate in the regression models would induce a bias (those with laxative use score higher in item GIDS-PD 1), we repeated the linear regression analysis for GIDS-PD constipation in participants who were not on regular laxatives (H&Y = 1, *n* = 67; H&Y = 2, *n* = 162; H&Y = 3, *n* = 27; H&Y = 4, *n* = 11) and observed similar results, with RBD prodromal history, but not H&Y and other variables, predicting GIDS-PD Constipation scores (*F*(6, 150) = 2.295, *p* = 0.038, *R*^2^ = 0.09).

### Longitudinal characterisation of GI scores

**Fig. 2:**
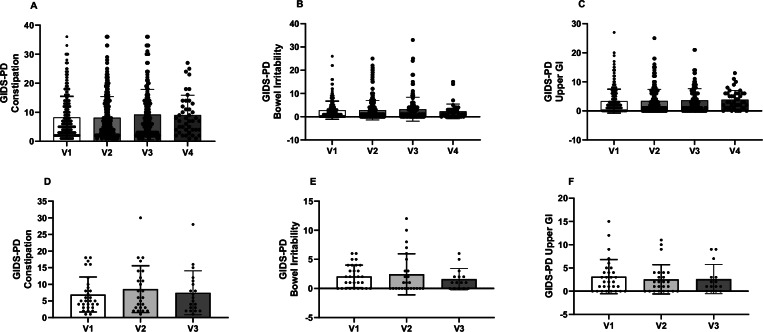
Longitudinal GIDS-PD domain scores in PD and RBD patients. No significant differences in GIDS-PD scores were observed over 4 visits spanning an average of 3-year follow-up in 245 PD participants (**A**–**C**) and over 2 year follow-up (**D**–**F**) in 25 RBD cases. GIDS-PD Gastrointestinal Dysfunction Scale–Parkinson’s disease, RBD REM Sleep Behaviour Disorder. Error bars represent SD.

A subset of participants completed the GIDS-PD longitudinally. 281 participants completed it twice, with an average interval of 1.6 (±0.7) years between timepoints. Of those, 144 participants completed it a third time, on average 2.8 (±0.9) years after visit 1 and 45 completed it a fourth time, on average 3.8 (±0.8) years after visit 1. Mixed-effects analysis showed a significant increase in PD motor severity (MDS-UPDRS-III) at each timepoint (score of 29.8, 33.8, 38.8 and 45.9 respectively; *F*(1.213, 132.6) = 39.32, *p* < 0.0001) as well as in Hoehn and Yahr stage (*F*(1.457, 192.3) = 27.43, *p* < 0.0001) and LEDD (328.3 mg, 550.0.mg, 631.8 mg and 777.9 mg respectively; (*F*(0.7315, 91.44) = 84.17, *p* < 0.0001). Mixed-effects models did not show significant differences in GIDS-PD scores between timepoints over an average of 4 years of follow-up (GIDS-PD Constipation, *F*(2.708, 412.6) = 2.142, *p* = 0.1009; GIDS-PD Bowel Irritability, *F*(2.668, 406.4) = 1.354, *p* = 0.2583; GIDS - PD Upper GI, *F*(2.864,433.4) = 0.3570, *p* = 0.7748, Mixed-effects models) (Fig. 2). Participants were then stratified according to PD duration (≤2.0 years from PD diagnosis at v1, *n* = 162; ≥2.01 years from PD diagnosis at v1, *n* = 119) to investigate whether longitudinal changes in GIDS-PD scores were more prominent in early or later disease. Mixed-effects models did not reveal significant differences in longitudinal GIDS-PD scores in either group (*p* > 0.2852).

A paired longitudinal analyses of 40 PD participants who were drug-naïve at baseline and started on dopaminergic medication at the subsequent follow-up visit (interval between visits 1.5 y ± 0.6), showed a significant decrease in bowel frequency scores (GIDS-PD item 1, *W* = 133, *p* = 0.0416), increase in straining (GIDS-PD item 2, *W* = 89.00, *p* = 0.0502) and no differences in dysphagia or gastric emptying scores (GIDS-PD item 9, *p* > 0.9999; GIDS-PD item 7, *p* = 0.07267, respectively) between the first and second visit.

25 iRBD cases completed the GIDS-PD longitudinally, after an average time interval of 1.1 (±0.1) years and 20 completed it a third time at 2.2 (±0.4) years follow-up. At the third time-point, 3 RBD participants had converted to PD and 1 to Lewy Body dementia. Mixed effects models did not show differences in GIDS-PD scores of iRBD participants between visits 1, 2 and 3 (0.4244 > *F* < 1.712, 0.5417 < *p* < 0.2026).

### Objective measurement of GID

**Fig. 3:**
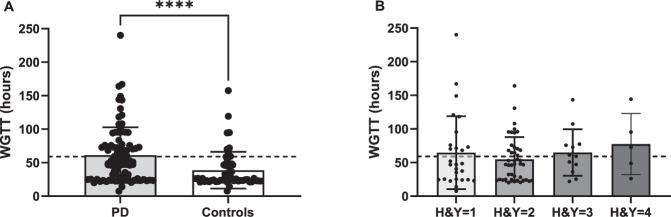
Whole gut transit time (in hours) in PD cases versus household controls and in PD cases stratified by H&Y stage. WGTT was significantly higher in PD cases compared to household controls (**A**). Within the PD cohort, there were no differences in WGTT across H&Y stages (**B**). Grid line depicts cut-off for delayed transit time (59 h). H&Y Hoehn and Yahr, PD Parkinson’s Disease, WGTT whole gut transit time. ****p ≤ 0.0001. Error bars represent SD.

Ninety-six participants with PD and 54 paired household controls ingested a blue food dye to measure whole gut transit time (WGTT). Ten PD cases, but no controls, took laxatives in the days following the blue dye ingestion and were excluded from the analysis. Eighty-six participants were included in the analysis (H&Y = 1, *n* = 27; H&Y = 2, *n* = 42; H&Y = 3, *n* = 12; H&Y = 4, *n* = 5). None reported discomfort following dye ingestion. There were no differences in the interval between the last normal stool and food dye ingestion between PD (0.4 ± 0.4 h) and controls (0.3 ± 0.4 h) (*W* = −300 *p* = 0.9788, Wilcoxon). WGTT was significantly higher in PD cases (62.9 ± 43.4 h) compared to household controls (36.6 ± 26.4 h) (*W* = −993.0, *p* < 0.001, Wilcoxon) (Fig. 3A). Thirty-eight PD cases (44.1%) and 8 controls (14.8%) were above normative cut-off for slow transit time (≥59 h)^20^, with the longest transit time recorded at 240 h in the PD group, despite the participant reporting daily emptying of bowels. There were no differences in WGTT across H&Y stages (Kruskal–Wallis test, *H*(4) = 2.932, *p* = 0.4022) (Fig. 3B).

Slower transit time was significantly correlated with higher GIDS-PD Constipation score (rho = 0.53, *p* < 0.001) and MDS-UPDRS constipation (item 1.1, rho = 0.32, *p* = 0.003), providing objective validation of the GIDS-PD as a tool to assess gut function in PD (Supplementary Fig. 1). WGTT correlated with age at visit (rho = 0.30, *p* = 0.005), LEDD (rho = 0.21, *p* = 0.049) and motor fluctuations (MDS-UPDRS Part IV, rho = 0.23, *p* = 0.036) but not with other MDS-UPDRS scores or other GIDS-PD scores. GIDS-PD constipation scores also were significantly associated with motor fluctuations (rho = 0.18, *p* = 0.036). There were no significant associations between WGTT and disease duration or cognitive scores. Partial correlations between WGTT and GIDS-PD Constipation scores withstood adjustment for age and LEDD (*r* = 0.31, *p* = 0.005). Between group comparison (Mann–Whitney *U* test) showed that PD patients with a high WGTT (≥59 h) had higher GIDS-PD Constipation scores compared to those with lower WGTT (*p* < 0.001), but no such differences were found for Upper GI subscores or abdominal fullness (GIDS-PD item 7), suggesting that the WGTT is more reflective of intestinal transit time rather than gastric emptying.

## Discussion

In this study, we formally characterised self-reported gastrointestinal dysfunction across disease stages, using the GIDS-PD. Our findings indicate that GID is common even in the prodromal phase of the disease (in patients with RBD), and although the prevalence of most GI symptoms increases with disease stage, a subset of participants with advanced disease did not report symptoms, suggesting that GI dysfunction is not a universal feature of the disease. Following adjustment for covariates, upper GI dysfunction was significantly associated with disease stage. However, our data indicate that constipation is not driven by disease stage or motor severity but is better explained by other factors such as a positive history of RBD and lack of exercise. The concept that constipation is not associated with PD severity is supported by previous studies. Rodriguez-Blazquez^21^ did not find significant correlations with the gastrointestinal score and motor scores of the SCOPA-AUT or H&Y in a sample of 387 people with PD. Using the Parkinson’s Progression Markers Initiative database, Pagano et al.^6^, found a significant but weak correlation between constipation and MDS-UPDRS-III score after adjusting for age (*r* = 0.10, *p* = 0.037). In the same study, dopamine transporter imaging did not show significant correlations between constipation score and dopaminergic pathology, leading the authors to suggest that constipation may be due to an impairment of non-dopaminergic pathways. Accordingly, Dai et al.^22^ also found that constipation was associated with axial symptoms (*r* = 0.19, *p* = 0.012), which are considered to be non-dopaminergic. One current theory in the aetiology and progression of PD is the ‘Body-first versus Brain-first’ hypothesis, which associates iRBD with a peripheral nervous system involvement subtype, characterised by autonomic damage and gut symptomatology before involvement of the CNS dopaminergic system^2^. Our adjusted analysis of GIDS-PD Constipation scores supports this hypothesis as it suggests that constipation is not associated with disease severity but with prodromal RBD history.

Congruently, a high prevalence of GI symptoms in the iRBD cohort was also observed, equivalent to PD stages H&Y 1 and 2. In a multi-centre study, Ferini-Strambi et al.^23^ found high prevalence of GID in 318 iRBD cases with significant differences in constipation, straining and sialorrhoea compared to controls^23^. Aguirre-Mardones et al. also found high prevalence of constipation (52%) and straining (73%) in a sample of 44 iRBD cases^24^. These prevalence figures are in keeping with those found in this study. The higher prevalence of several GIDS-PD symptoms in the iRBD cohort compared to early PD cohort supports the hypothesis given that the iRBD group is likely composed of ‘body-first’ prodromal PD whereas the other PD groups are likely to include a combination of cases with ‘brain-first’ PD and ‘body-first’ PD. Moreover, when we stratified all PD participants together by RBD symptoms prior to PD diagnosis, those who were RBD positive had significantly higher GIDS-PD constipation scores and prodromal RBD was a strong predictor of GIDS-PD scores.

It is unlikely that the lower GIDS-PD Constipation scores in the early PD group compared to the iRBD group are explained by the introduction of dopaminergic medication. Dopaminergic medication has been consistently associated with an increase in constipation (e.g., decreased bowel frequency and increased gastrointestinal transit time)^5,25,26–29^, and our subanalysis of drug naïve PD cases showed a deterioration of constipation symptoms after PD medication introduction, confirming previous findings. Thus introduction of dopaminergic medication is unlikely to fully explain the drop in prevalence in early PD when compared to the iRBD. Of note, we also found an association between with motor fluctuations and both higher GIDS-PD constipation scores and higher WGTT, indicating that constipation may impact on symptom management, most likely through the inhibitive effect on levodopa absorption.

In keeping with the adjusted regression analysis for GIDS-PD Constipation, longitudinal analysis of GIDS-PD Constipation scores suggested minimal change over time. We also did not observe a significant increase in Bowel Irritability and Upper GI subscores over 4 years of follow-up. Constipation is typically thought to worsen with disease duration due to progression of PD pathology, but our data (cross-sectional regression analysis and longitudinal analysis of self-reported GI symptoms and WGTT) suggests that constipation is a feature of a subset of people with Parkinson’s. Our findings have important implications for patient stratification in clinical studies and clinical trials, as well as for the clinical management of gut symptoms in PD through allowing health teams to identify those at risk of constipation and promoting better understanding of protective factors.

It is possible that the lack of an observed association between constipation severity and disease stage is attributable to the increased prevalence of daily laxative intake in higher disease stages, however, the WGTT substudy only included participants that did not take laxatives and did not show increase in transit time across stages. Furthermore, linear regression analysis in the subset of people who did not take laxatives confirmed the main regression analysis findings, with prodromal RBD, but not H&Y, being the only significant predictor of GIDS-PD constipation score.

Only one other longitudinal study has found similar results to ours. Edwards et al.^7^ followed a small heterogenous sample of 56 PD cases (H&Y = 1–5) over 18 months and found an increase in frequency and severity of constipation, but not in sialorrhoea, dysphagia, heartburn, bloating or nausea as measured by their custom-made (unvalidated) GID survey. They also did not see a difference in total GI scores or the development of new GI symptoms over the follow-up period, which they attributed to a lack of significant change in PD severity and short follow-up period. Conversely, Stanković et al.^10^ followed 86 de novo PD cases and found that all SCOPA-AUT items regarding gastrointestinal dysfunction significantly increased over 3 years, except for dysphagia. Erro et al.^9^ followed 61 de-novo untreated PD cases and found that sialorrhoea, swallowing difficulties, nausea, weight loss and constipation, as measured by the Non-Motor Symptom Scale, increased significantly at a 4-year follow-up. However, no PD staging was available, and the follow-up included the introduction of dopaminergic medication which is known to worsen constipation^5,25,26–29^. It is thus possible that the discrepancies between previous studies and ours may be attributable to the fact that 211 of our 245 participants were already on dopaminergic medication at visit 1 thus minimising the dopaminergic effect seen in other studies, other sample characteristics (e.g. over-representation of a given subtype such as body-first PD), as well as the lack of adjustment for covariates in other studies. A key aspect of this study is that it highlights the importance of confounding factors on influencing the relationship between GID and disease stage. In addition to prodromal RBD, caffeine intake, and exercise were identified as factors associated with GIDS-PD scores. Exercise has previously been associated with improvement of constipation in PD^30^, a finding reinforced by our results, with important implications for self-management. Coffee intake was associated with less bowel irritability scores in keeping with studies which attribute a protective effect of caffeine to motor and non-motor symptoms of PD^29,31^ but which could also be attributable to avoidance behaviour, as people with bowel irritability tend to avoid caffeinated drinks. In our study, anticholinergic drug use was also associated with increased GIDS-PD Bowel Irritability and Upper GI scores, likely due to side-effects such as gastroesophageal reflux, nausea, vomiting, constipation, bloating, abdominal pain, and weight loss^32–34^.

In keeping with the findings of the GIDS-PD analyses, we did not find differences in transit time across H&Y stages, which supports the premise that constipation is a relatively stable feature in PD. Similarly, studies using radio opaque markers (ROMs) have reported differences between controls and PD cases but have not shown significant correlations with PD severity^16,35,36^. The average transit time in our study also corroborated previous literature using ROMs (89–108 h for PD cases)^16,35,36^. Importantly, the GIDS-PD Total and Constipation scores correlated significantly with WGTT, providing objective validation of the GIDS-PD as a tool to assess gut function in PD and outperforming correlation strength between other psychometric measures of constipation and ROMs^11–13^.

The presentation of difficulty in swallowing in iRBD is another interesting finding of this study. Dysphagia is classically considered to be a complication of advanced PD and there are few studies investigating its prevalence in early stages when it may be an indicator of an alternate diagnosis^37^. One study has reported dysphagia being present 2 years before PD diagnosis^38^. Similar to our results, Verbaan et al.^39^ noted a difficulty swallowing frequency of 34% in H&Y = 1 & 2 whilst Erro et al.^9^ observed a prevalence of 11.1% in early untreated PD cases. A study by Konings et al., which identified GI predictors of PD onset in a large US based nationwide medical record network, showed strong association between dysphagia and PD onset (RR 2.27, 95% CI 2.10–2.45)^40^. However, difficulty swallowing and/or dysphagia can be present in older populations without neurological disorders and partly attributed to factors such as age, medication and heartburn^41,42^.

Limitations of this study must be noted. In spite of its comprehensive nature, the GIDS-PD relies on self-report and it is therefore possible that GI symptom frequency and severity were under reported. However, the GID-PD Constipation subscore and WGTT showed a strong significant correlation suggesting that the GIDS-PD is a reliable instrument. Group characteristics were also an important limitation. The iRBD group was predominantly male (only 5% women) which might contribute to a lower observed frequency of GI symptoms as females are known to have higher prevalence of GID in the general population^43,44^. In the PD cohort, history of RBD was self-reported and not confirmed by PSG, as such iRBD history might be underreported as patients with mild severity of symptoms and/or who do not have a sleep partner are less likely to report it^45^. Lastly, the longitudinal follow-up of only 4 years provides limited information on how GI symptoms evolve over the disease course, however, we found a significant increase in UPDRS-III scores, H&Y as well as LEDD over this period thus the lack of progression in GID scores cannot not be attributed to lack of PD motor progression within this time frame. Future studies with larger sample sizes and longer follow-up period that adjust for relevant confounders will add to our understanding of GID across PD stages and its relationship with disease progression.

In conclusion, this study shows that gut dysfunction is common in PD and occurs from the earliest stages of the disease process (before the onset of motor symptoms) but is heterogeneous. GID is not a universal feature of PD, even in very late-stage disease, but rather occurs in a subset of cases. Cross-sectional and longitudinal analysis of both objective and self-reported measures of gut function suggest that constipation is not associated with disease severity and could thus represent a PD phenotype, whereas upper GI symptoms increase incrementally with disease stage. Moreover, our findings have important implications for patient stratification in clinical studies and clinical trials, as well as for the clinical management of gut symptoms in PD by allowing healthcare teams to identify those at risk of constipation and promoting engagement with protective factors (e.g., physical exercise).

## Methods

### Participants

This study was approved by the Cambridgeshire 3 Research Ethics Committee (08/H0306/26). PD and iRBD participants were recruited from the Cambridge Parkinson’s Disease Research Clinic and the Discovery Cohort study (Targeting the early pathological pathways in Parkinson’s Disease)^46^, respectively. All participants were approached when they came to their regular research appointment. PD cases met UK Parkinson’s Disease Society Brain Bank diagnostic criteria for idiopathic PD, and exclusion criteria included a diagnosis of dementia according to MDS criteria^47^ and the presence of other neurodegenerative disorders. iRBD cases were diagnosed by a sleep physician and confirmed by polysomnography. All iRBD patients had been reviewed by a neurologist to exclude phenoconversion to PD or related synucleinopathies using diagnostic criteria at the time of study^48^. Controls were spouses of iRBD and PD participants and the only exclusion criterion was the presence of neurodegenerative disorder. Written informed consent was obtained from all participants. Participants with gastrointestinal conditions were not excluded as the aim was to capture a comprehensive overview of GI symptoms in PD.

### Data collection

Clinical assessments coincided with participant’s regular research appointments and were performed between December 2019 and April 2024 at the research clinic or as home visits for those with accessibility issues. Longitudinal assessments were conducted at 12 to 24 months intervals, according to the scheduling of their research clinic appointments. A trained clinician (neurologist/neuropsychologist) conducted clinical assessments. Participants were assessed using the MDS-UPDRS (during ‘on’ state if medicated for PD) and the Hoehn and Yahr (H&Y) scale. In people with PD, history of RBD symptoms prior to PD diagnosis was recorded via clinical assessment. Levodopa equivalent daily dose (LEDD) was calculated according to an adapted version of the Tomlinson formula^49^. Anticholinergic medication and opiate-based drug use was recorded. Anticholinergic burden was calculated using an online calculator adapted from Scottish Intercollegiate Guidelines Network (available at: https://www.acbcalc.com/). Montreal Cognitive Assessment (MoCA)^50^ was administered to assess cognitive function. Gastrointestinal symptoms were assessed with the GIDS-PD^19^, a Likert self-report scale composed of 12 items pertaining to 3 domains: Constipation (frequency of bowel movements, straining, hardness of stools, and incomplete evacuation), Bowel Irritability (abdominal pain, abnormal increase in stool passing, distension/bloating, and weight loss), and Upper GI symptoms (dysphagia, sialorrhea, heartburn, and nausea). Supplementary items 1a (measures taken to increase bowel movement frequency), 1b (duration of constipation), A (diet), B (water intake), C (caffeinated drinks intake), D (exercise), E (smoking), and F (medical diagnosis of GID) are not used for scoring but provide important additional information.

Gut transit time was measured as the length of time, in hours, between ingestion of a blue food dye (PME^©^ sky blue) and first appearance of blue stool^18^ (Blue Poop Challenge). Participants were asked to ingest 20 drops of dye (approximately 1 mL), mixed with a liquid of their choice, within 2 h of their last defecation. Its ingredients are considered safe for human consumption^51,52^. Participants were asked to avoid laxatives after ingesting the blue food dye and when this was not possible due to discomfort, laxative use was annotated and the participant excluded from analysis. The BPC was performed only once per participant.

### Data analysis

Data analyses were performed using SPSS version 29.0 and Graphpad Prism version 9. Results are presented as mean ± standard deviation (SD). All analyses were two-tailed, with significance considered at *p* < 0.05 for unadjusted comparisons. Any missing item on the GIDS-PD invalidated its total score and domain scores. Shapiro–Wilk’s test did not confirm distribution normality for continuous variables therefore Kruskal–Wallis or Wilcoxon signed-rank tests were used for between-group comparisons of continuous variables, as appropriate, and Chi-square (*χ*^2^) analysis were used for comparisons of dichotomous variables. Bonferroni correction for multiple tests were used for pairwise comparisons. Spearman’s correlation coefficient was used to correlate GIDS-PD scores with other clinical measures and WGTT. A base linear regression model was created to assess the predictive role of age, sex and H&Y stage on GIDS-PD domain scores (model 1). Then, a backward stepwise approach was used until best model fit was achieved. Covariates included age at visit, sex, medication use (LEDD, opioids, anticholinergics), history of prodromal RBD and relevant lifestyle variables (diet, water intake, smoking and exercise level).

## Supplementary information

Supplementary table 1 and Supplementary figure 1

## Data Availability

Anonymized data are available upon reasonable request by any qualified investigator.
