# Supplementary material for: The profile of gastrointestinal dysfunction in prodromal to late-stage Parkinson’s disease
Source: NPJ Parkinsons Dis. 2025 May 11;11:123. doi: 10.1038/s41531-025-00900-9 (PMC12065915; doi:10.1038/s41531-025-00900-9)
Supplement: Supplementary file 1 — Supplementary table 1 and Supplementary figure 1 [file 41531_2025_900_MOESM1_ESM.pdf]

GIDS-PD Constipation	Model 1 R^2=0.05 (F(3, 377)=6.095, *p*<0.001)			Model 2 R^2=0.15 (F(12, 223)=3.280, *p*<0.001)		
	β	95% CI	*p*-value	β	95% CI	*p*-value
**MDS-UPDRS-III**	0.07	0.03 – 0.12	0.003*	0.04	-0.03 – 0.11	0.239
**Age at visit**	0.02	-0.06 – 0.10	0.652	0.03	-0.08 – 0.13	0.630
**Sex** (Female)	2.02	0.55 – 3.48	0.007*	1.77	-0.13 – 3.66	0.068
**Exercise** (3+ times a week)				-1.19	-2.29 – -0.08	0.036*
**Anticholinergic use**				2.42	-0.21 – 5.05	0.071
**Opioids**				-3.84	-7.88 – 0.19	0.062
**Prodromal RBD**				2.17	0.26 – 4.08	0.026*
**LEDD**				0.00	0.0 – 0.0	0.119
**Vegetarian diet**				3.36	-0.64 – 7.36	0.099
**Water** (4+ a day)				0.34	-1.12 – 1.80	0.646
**Low caffeine** (<1 caffeinated drink a day)				0.54	-1.75 – 2.82	0.647
**Smoking** (never smoked)				-0.60	-2.42 – 1.22	0.516
**GIDS-PD Bowel Irritability**	**Model 1** R^2=0.04 (F(3, 374)=4.517, *p*=0.004)			**Model 2** R^2=0.18 (F(12, 219)=4.012, *p*<0.001)		
	β	95% CI	*p*-value	β	95% CI	*p*-value
**MDS-UPDRS-III**	0.03	0.0 – 0.06	0.027*	0.02	-0.02 – 0.06	0.308
**Age at visit**	-0.02	-0.07 – 0.02	0.298	-0.04	-0.10 – 0.02	0.232
**Sex** (Female)	1.28	0.44 – 2.11	0.003*	0.97	-0.10 –2.05	0.076
**Caffeine** (4+cups a day)				-1.01	-1.71 – -0.30	0.005*
**Smoking** (Never smoked)				-0.95	-2.00 – 0.09	0.074
**Low Exercise** (less than once a week)				0.87	-0.23 – 1.97	0.119
**Anticholinergic use**				1.95	-0.43 – 3.47	0.012*
**Opioids**				-0.56	-2.85 – 1.73	0.63
**LEDD**				0.00	0.0 – 0.0	0.069
**Vegetarian diet**				-1.02	-3.28 – 1.24	0.377

**Water intake** (4+ a day)				0.70	-0.13 – 1.54	0.097
**RBD history**				0.09	-1.00 – 1.18	0.876
**GIDS-PD Upper GI**	**Model 1** R^2=0.10 (F(3, 378)=13.576, *p*<0.001)			**Model 2** R^2=0.22 (F(9, 228)=6.944, *p*<0.001)		
	β	95% CI	*p*-value	β	95% CI	*p*-value
**MDS-UPDRS-III**	0.74	0.05– 0.10	<0.001*	0.08	0.05 – 0.12	<0.001*
**Age at visit**	0.02	-0.02 – 0.06	0.408	0.02	-0.04 – 0.07	0.552
**Sex (Female)**	0.39	-0.39 – 1.17	0.331	0.34	-0.67 – 1.35	0.509
**LEDD**				0.0	0.0 – 0.003	0.026*
**Anticholinergic use**				1.89	-0.55– 3.22	0.006*
**Water intake** (4+ a day)				0.39	-0.38 – 1.17	0.321
**Caffeine** (4+cups a day)				-0.20	-0.85 – 0.46	0.559
**Exercise** (3+ times a week)				0.61	-0.03 – 1.20	0.041*
**RBD history**				0.50	-0.52 – 1.51	0.336

Supplementary table 1. Regression models for GIDS-PD domain scores. Significance threshold *p*<0.05, represented by *. β - beta coefficient; CI, Confidence Interval; PD - Parkinson's disease; GIDS-PD – Gastrointestinal Dysfunction Scale for Parkinson's disease; LEDD- Levodopa equivalent daily dose; MDS-UPDRS-III: Movement Disorders Society – Unified Parkinson's Disease Rating Scale Motor Scale.

Supplementary Figure 1. Correlation between WGTT (in hours) and GIDS-PD Constipation domain score.

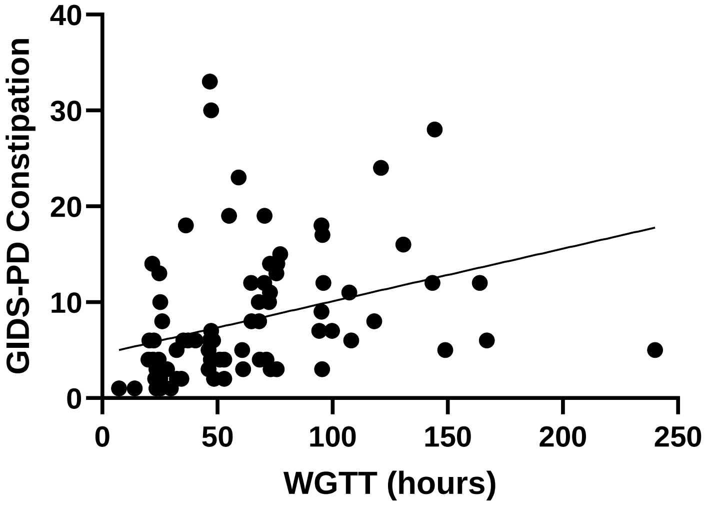

WGTT correlated positively with GIDS-PD Constipation in participants with PD (rho=0.53, $p<0.001$; n=86). WGTT – whole gut transit time. GIDS-PD – Gastrointestinal Dysfunction Scale for Parkinson’s Disease.
